# Plasma from septic shock patients induces loss of muscle protein

**DOI:** 10.1186/cc10475

**Published:** 2011-09-29

**Authors:** Hieronymus WH van Hees, Willem-Jan M Schellekens, Marianne Linkels, Floris Leenders, Jan Zoll, Rogier Donders, PN Richard Dekhuijzen, Johannes G van der Hoeven, Leo MA Heunks

**Affiliations:** 1 Department of Pulmonary Diseases, Radboud University Nijmegen Medical Centre, Geert Grooteplein-Zuid 10, 6525 GA, Nijmegen, The Netherlands; 2 Department of Anesthesiology, Radboud University Nijmegen Medical Centre, Geert Grooteplein-Zuid 10, 6525 GA, Nijmegen, The Netherlands; 3 Department of Medical Microbiology, Radboud University Nijmegen Medical Centre, Geert Grooteplein-Zuid 10, 6525 GA, Nijmegen, The Netherlands; 4 Department of Epidemiology, Biostatistics and HTA, Radboud University Nijmegen Medical Centre, Geert Grooteplein-Zuid 10, 6525 GA, Nijmegen, The Netherlands; 5 Department of Intensive Care Medicine, Radboud University Nijmegen Medical Centre, Geert Grooteplein-Zuid 10, 6525 GA, Nijmegen, The Netherlands

## Abstract

**Introduction:**

ICU-acquired muscle weakness commonly occurs in patients with septic shock and is associated with poor outcome. Although atrophy is known to be involved, it is unclear whether ligands in plasma from these patients are responsible for initiating degradation of muscle proteins. The aim of the present study was to investigate if plasma from septic shock patients induces skeletal muscle atrophy and to examine the time course of plasma-induced muscle atrophy during ICU stay.

**Methods:**

Plasma was derived from septic shock patients within 24 hours after hospital admission (n = 21) and healthy controls (n = 12). From nine patients with septic shock plasma was additionally derived at two, five and seven days after ICU admission. These plasma samples were added to skeletal myotubes, cultured from murine myoblasts. After incubation for 24 hours, myotubes were harvested and analyzed on myosin content, mRNA expression of E3-ligase and Nuclear Factor Kappa B (NFκB) activity. Plasma samples were analyzed on cytokine concentrations.

**Results:**

Myosin content was approximately 25% lower in myotubes exposed to plasma from septic shock patients than in myotubes exposed to plasma from controls (*P *< 0.01). Furthermore, patient plasma increased expression of E3-ligases Muscle RING Finger protein-1 (MuRF-1) and Muscle Atrophy F-box protein (MAFbx) (*P *< 0.01), enhanced NFκB activity (*P *< 0.05) and elevated levels of ubiquitinated myosin in myotubes. Myosin loss was significantly associated with elevated plasma levels of interleukin (IL)-6 in septic shock patients (*P *< 0.001). Addition of antiIL-6 to septic shock plasma diminished the loss of myosin in exposed myotubes by approximately 25% (*P *< 0.05). Patient plasma obtained later during ICU stay did not significantly reduce myosin content compared to controls.

**Conclusions:**

Plasma from patients with septic shock induces loss of myosin and activates key regulators of proteolysis in skeletal myotubes. IL-6 is an important player in sepsis-induced muscle atrophy in this model. The potential to induce atrophy is strongest in plasma obtained during the early phase of human sepsis.

## Introduction

Skeletal muscle weakness frequently develops in patients admitted to the intensive care unit (ICU), with a reported incidence between 25% and 60% after more than one week of mechanical ventilation [1]. ICU-acquired weakness of the respiratory muscles is associated with prolonged weaning and weakness of peripheral muscles is associated with prolonged rehabilitation [2]. Mortality in patients with ICU-acquired weakness is higher than in ICU patients without weakness [3]. In addition, in survivors the consequences of ICU-acquired muscle weakness may persist for more than one year after ICU discharge [2]. Besides the devastating physical and psychological effects to patients and their family, ICU-acquired weakness has a major economical impact.

Among other factors, sepsis is known to be an important predictor for the development of ICU-acquired muscle weakness [4]. In septic patients, weakness may originate from any point between the central nervous system and the contractile proteins (for review [5]). The effect of sepsis on contractile proteins has received much attention [6,7]. For instance, Tiao *et al*. [6] found that in septic rats, skeletal muscle protein breakdown was increased due to enhanced activity of the proteolytic ubiquitin-proteasome pathway. However, it is unknown whether ligands in human septic plasma can activate the ubiquitin-proteasome pathway and induce loss of myosin. This is of relevance as it will help to understand the importance of systemic components compared to intrinsic muscle factors, such as disuse, in sepsis-induced muscle atrophy. Previous studies, indeed, have indicated that cytokines and bacterial cell wall components induce muscle proteolysis in sepsis [8,9], but specific pathways have not been evaluated. Therefore, the objective of the current study was to investigate whether plasma from patients with septic shock induces loss of myosin and whether this is associated with activation of the ubiquitin-proteasome pathway. We exposed non-diseased cultured skeletal muscle to plasma from patients with septic shock. It was hypothesized that septic plasma induces loss of muscle proteins in highly differentiated skeletal muscle cells. In a follow-up study we investigated the time course of the atrophic response induced by plasma obtained from patients admitted with septic shock. In addition, we explored the relation between myosin loss and plasma levels of inflammatory cytokines.

## Materials and methods

### Experimental design

Two separate sets of experiments were performed. The first set of experiments was conducted to establish whether plasma in the early phase of septic shock induces muscle proteolysis and atrophy. To this end, cultured skeletal muscle myotubes were incubated with either plasma from patients with septic shock obtained within 24 hours of ICU admission or plasma from healthy subjects (controls). After 24 hours of exposure to plasma, skeletal myotubes were harvested for biochemical analysis, including myosin heavy chain content and expression of E3-ligases. The activity of nuclear factor kappa b (NFκB) was measured after one hour of exposure to plasma.

The second set of experiments was conducted to investigate if the ability of plasma from septic shock patients to induce muscle atrophy changes during the course of ICU admission and if a correlation exists between inflammatory cytokines and loss of myosin. Hence, plasma was derived at different time points during an ICU stay of patients with a septic shock and the ability of each plasma sample to induce proteolysis and atrophy in differentiated muscle cells was examined. In addition, plasma levels of cytokines were determined at each time point.

### Study population

Twenty-one patients with established septic shock, according to the 2001 International Sepsis Definitions Conference [10], were included in the first set of experiments. Blood was withdrawn from the indwelling arterial catheter within 24 hours of ICU admission. In 12 control subjects blood was obtained through venapuncture.

For the second set of experiments, additional blood samples were obtained from 9 of the 21 septic shock patients at Days 2, 5 and 7 of the ICU stay. The institutional review board, the medical ethics committee, approved the current study and waived the need for informed consent.

### Muscle culture

Muscle cells, C2C12 myoblasts, were cultured into myotubes according to previously described methods [11]. Pilot studies showed stable myosin content in myotubes that differentiated for seven days and that plasma from healthy subjects does not affect myosin content in these myotubes. See Additional file 1 for data and more details.

### Analysis of myosin content

Myosin content in myotubes was determined by Western blotting according to previous described methods. See Additional file 1 for more details.

### Analysis of NFκB activity

NFκB is a ubiquitous transcription factor for a variety of cytokines. The DNA binding activity of NFκB has been shown to be highest within several hours after exposure to different stimuli [12,13], which was confirmed by pilot-experiments in our lab. Therefore, in the current study NFκB activity was measured one hour after incubation of the myotubes with plasma.

NFκB DNA binding activity was determined by electrophoretic mobility shift assay (EMSA) conform previously described methods [14]. See Additional file 1 for details.

### Analysis of E3-ligase mRNA and ubiquitinated myosin levels

Expression of muscle specific E3-ligases, Muscle RING Finger protein-1 (MuRF-1) and Muscle Atrophy F-box protein (MAFbx) and ubiquitination of myosin was analyzed, conforming to previously described methods [15], see Additional file 1 for details.

### Cytokines

Plasma levels of IL-6, IFN-γ, TNF-α and IL-1β were measured by enzyme-linked immunosorbent assay (ELISA) (for IL-6, IFN-γ and TNF-α ELISA kits from Sanquin Reagents, Amsterdam, The Netherlands, for IL-1β Quantikinekit from R&D Systems, Minneapolis, MN, USA). To further examine the role of IL-6, we first studied myosin content in myotubes that were exposed to septic shock plasma containing an antibody directed against human IL-6 (R&D Systems). In addition, we studied myosin content in myotubes exposed to control plasma containing different concentrations of recombinant human IL-6 (Invitrogen, Carlsbad, CA, USA).

### Data analysis

Data are presented as means ± standard deviation. The statistical significances of differences regarding myosin content, MuRF-1 and MAFbx expression between myotubes exposed to plasma from controls and patient plasma were analyzed by performing Student *t*-tests. Differences regarding NFκB activity and cytokine concentrations were statistically tested by performing a Mann Whitney U test. Sample size calculations for the follow-up study showed that nine patients should be sufficient to detect a 25% reduction of myosin content with a standard deviation of 20%, 80% probability and an alpha level of 0.05. One-way ANOVA with *post-hoc *Bonferroni's multiple comparison testing was used to evaluate whether myosin content, MuRF-1 and MAFbx expression and cytokine levels at each day were statistically different from control. Repeated measures one-way ANOVA with *post-hoc *Bonferroni's multiple comparison testing was performed to analyze differences between Day 0 and subsequent days. The relation between myosin and IL-6 levels was explored using a linear model estimated by Generalized Least Squares to allow for correlations between the measurements caused by repeatedly measuring the same subjects; an unstructured correlation matrix was used to model this dependence. Since IL-6 levels were not normally distributed, log transformation on these values was applied. *P*-values below 0.05 were considered significant.

## Results

### Patient characteristics

Patient characteristics are shown in Table 1. All patients met septic shock criteria. Thirteen of the 21 patients received one bolus of 100 mg hydrocortisone before blood withdrawal. Additional characteristics of patients included in the second set of experiments are shown in Additional file 1, Table S1. None of the control subjects reported any significant past medical history or current use of prescribed medication.

**Table 1:** Subject characteristics

	Control (n = 12)	Septic shock (n = 21)
Age (yrs)	59 ± 3	64 ± 3
Male (%)	75% (9/12)	81% (17/21)
APACHE score	NA	21 ± 2
Source of septic shock (%)		
Pulmonary		14% (3/21)
Gastrointestinal		38% (8/21)
Pancreatitis	NA	10% (2/21)
Urinary tract		5% (1/21)
Miscellaneous		33% (7/21)
Past medical history (%)		
COPD		19% (4/21)
Carcinoma	NA	38% (8/21)
Auto-immune disease		19% (4/21)
Diabetes		19% (4/21)

### Effects of early septic shock

#### Myosin content

**Figure 1:**
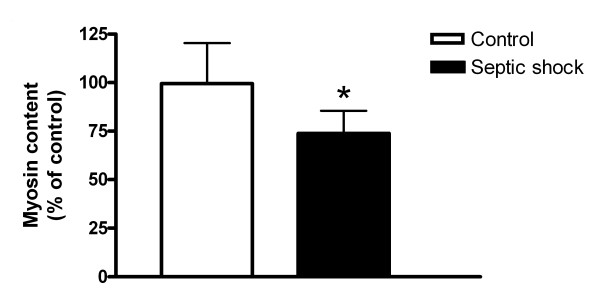
**Effect of septic shock plasma on myosin content**. Myosin content in myotubes that were either exposed to plasma from controls (n = 12) or plasma from patients with a septic shock (n = 21). *P < 0.01 vs. controls.

Myosin content in skeletal myotubes that were exposed to plasma from septic shock patients was approximately 25% lower than in myotubes that were exposed to plasma from controls (*P *< 0.01, Figure 1). In addition, the representative Western blot (Additional file 1, Figure S3) shows that tubulin content was not affected by septic plasma, excluding the involvement of a general protein loss. Furthermore, it demonstrates that myosin could not be detected in cell medium (experimental plasma), also excluding cell lysis (Additional file 1, Figure S4). Discrimination between patients that did and did not receive steroids prior to blood withdrawal shows that plasma samples from both groups induce a significant and similar myosin loss in myotubes compared to controls (Additional file 1, Figure S5).

#### NFκB activity and activation of the ubiquitin-proteasome pathway

**Figure 2:**
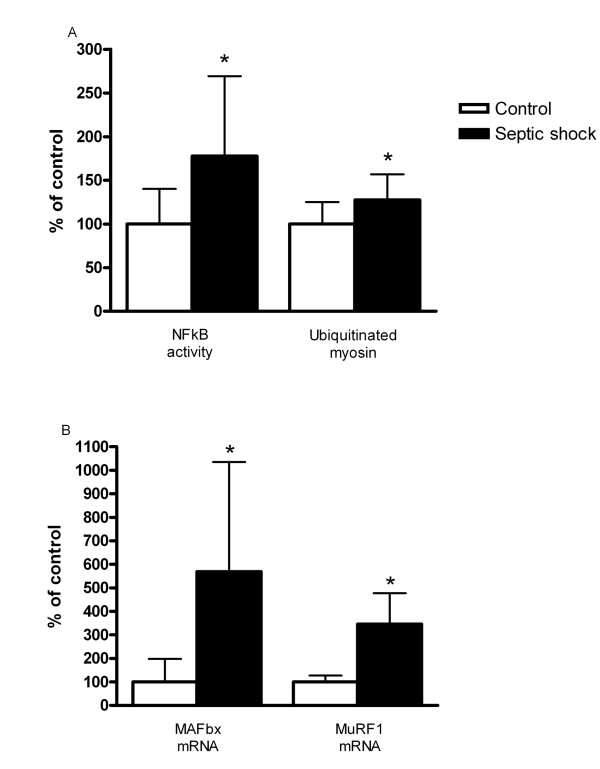
**Effect of septic shock on NFkB activity, ubiquitinated myosin and E3-ligase expression**. **A**) Effect of septic shock plasma on NFkB activity and ubiquitinated myosin. Nuclear Factor Kappa B (NFκB) activity in myotubes that were exposed for one hour to either plasma from controls (n = 6) or to plasma from patients with septic shock (n = 6) and ubiquitinated myosin per total myosin levels in myotubes after incubation for 24 hours with either plasma from controls (n = 12) or plasma from patients with septic shock (n = 12). *P < 0.05 vs. controls. **B**) Effect of septic shock plasma on E3-ligase expression. Muscle Atrophy F-box protein (MAFbx) and Muscle RING Finger protein-1 (MuRF-1) expression in myotubes after incubation for 24 hours with either plasma from controls (n = 5) or plasma from patients with septic shock (n = 14). *P < 0.05 vs. controls.

We measured NFκB activity as it is a key regulator of the inflammatory response and increased NFκB activity has been associated with activating the ubiquitin-proteasome pathway and inducing muscle atrophy [16]. Figure 2A shows that NFκB activity is significantly increased upon exposure to septic plasma (*P *< 0.05), see Additional file 1, Figure S6 for a representative Western blot.

The E3 ligases, MuRF-1 and MAFBx are key regulating enzymes of the proteolytic ubiquitin-proteasome pathway [17]. The mRNA levels of MAFbx and MuRF-1 were significantly higher in myotubes exposed to septic plasma (Figure 2B).

Finally, exposure to septic shock plasma increased ubiquitination of myosin (*P *< 0.05, Figure 2A).

### Effects of prolonged septic shock

Because the current data demonstrate that plasma from patients with septic shock induces atrophy at ICU admission, we performed additional experiments to follow this atrophic response in the course of ICU stay.

#### Myosin content

**Figure 3:**
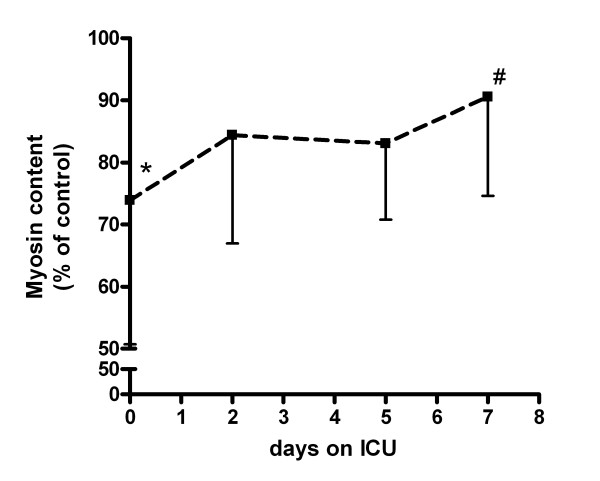
**Effect of septic shock plasma to myosin content during ICU stay**. Myosin content in myotubes that were exposed for 24 hours to plasma from healthy controls (n = 12) and plasma derived from patients with septic shock (n = 9) at admission (Day 0) and two, five and seven days after admission to the intensive care unit (ICU). *P < 0.05 vs. controls. #*P *< 0.05 vs. Day 0.

Figure 3 shows that plasma of patients with septic shock induced the strongest atrophic response at the first day of ICU admission. Exposure of myotubes to plasma obtained at Day 2 and Day 5 after ICU admission also resulted in lower reduced myosin content compared to control, but these differences were borderline statistically significant (*P *= 0.08 and *P *= 0.05 respectively). Myosin content in myotubes that were exposed to plasma obtained after seven days of ICU care was not significantly different from control (*P *= 0.28). Myosin content at Day 7 was significantly higher than at Day 0 (*P *< 0.01).

#### E3-ligase expression

**Figure 4:**
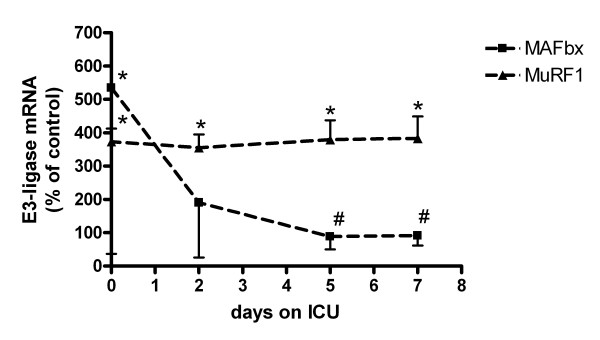
**Effect of septic shock plasma on E3-ligase expression during ICU stay**. Muscle Atrophy F-box protein (MAFbx) and Muscle RING Finger protein-1 (MuRF-1) expression in myotubes that were exposed for 24 hours to plasma from healthy controls (n = 5) and plasma derived from patients with septic shock (n = 9) at admission (Day 0) and two, five and seven days after admission to the intensive care unit (ICU). *P < 0.01 vs. controls. #*P *< 0.05 vs. Day 0.

MAFbx expression was highest in myotubes that were exposed to plasma taken at the first day of ICU admission (Figure 4). Plasma taken beyond that day did not significantly enhance MAFbx expression. MAFbx expression at Day 5 and Day 7 were significantly lower than at Day 0. In contrast, MuRF-1 expression was enhanced in myotubes by plasma obtained up to Day 7 after ICU admission (Figure 4).

#### Cytokines

**Figure 5:**
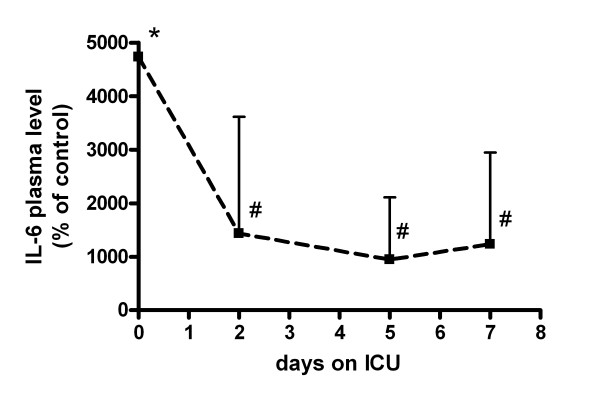
**Effect of septic shock on plasma IL-6 levels during ICU stay**. Interleukin-6 (IL-6) levels in plasma from controls (n = 9) and from patients with septic shock (n = 9) taken at admission (Day 0) and two, five and seven days after admission to intensive care unit (ICU). *P < 0.01 vs. controls. #*P *< 0.05 vs. Day 0.

**Figure 6:**
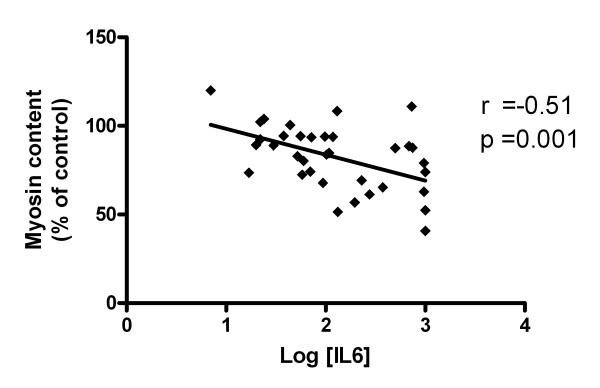
**Relation between IL-6 plasma levels and sepsis induced myosin loss**. Correlation between plasma Interleukin-6 (IL-6) levels and myosin content in myotubes exposed to plasma from septic shock patients. Log: common logarithm.

Since cytokines are known to induce proteolysis in muscle cells, we measured IL-1β, IL-6, IFN-γ and TNF-α levels in the plasma samples that were used in the experiments described above. Upon admission to the ICU, IL-6 levels were approximately 50-fold higher in plasma from patients with septic shock compared to controls (*P *< 0.001, Figure 5). Although IL-6 levels decreased rapidly during hospitalization, levels at Days 2, 5 and 7 were still approximately 10-fold higher than in controls (*P *= 0.05, *P *= 0.09 and *P *= 0.10 respectively), but significantly lower than at Day 0 (*P *< 0.01). Correlation statistics showed a significant negative association between myosin content and plasma IL-6 levels (*P *= 0.001, Figure 6).

IFN-γ, TNF-α and IL-1β levels were below the detection limit (respectively 8, 3 and 8 pg/ml) for all controls and for seven of nine patients with septic shock.

**Figure 7:**
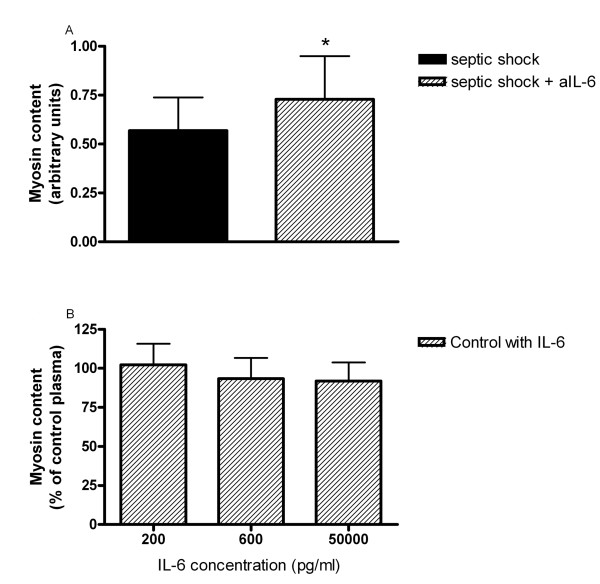
**Effect of anti-IL6 on sepsis induced myosin loss and recombinant IL-6 on myosin content**. **A**) Effect of anti-IL6 on sepsis induced myosin loss. Myosin content in myotubes that were exposed to plasma from septic shock patients (n = 10), with and without addition of anti-interleukin-6 (a-IL-6, 100 ng/ml). * *P *< 0.05. **B**) Effect of recombinant IL-6 on myosin content. Myosin content in myotubes that were exposed to plasma from controls (n = 10) with addition of IL-6 in different concentrations.

As these data suggest a causative role of plasma IL-6 in inducing muscle atrophy during septic shock, we performed two additional experiments. First, we examined the effect of blocking IL-6 in septic shock plasma on the atrophic response of skeletal myotubes. Figure 7A demonstrates that the addition of anti-IL-6 (100 ng/ml) to plasma from septic shock patients results in an approximately 25% higher myosin content in skeletal myotubes, see Additional file 1, Figure S7 for a representative Western blot. In addition, we examined the effect of elevated IL-6 concentration in plasma from controls on the atrophic response of skeletal myotubes. Figure 7B shows that addition of physiological IL-6 concentrations (that is, 200 and 600 pg/ml, the mean IL-6 plasma levels in septic patients at respectively Day 2 and Day 0) and supra-physiological (50 ng/ml) concentrations of IL-6 to plasma from controls did not induce an atrophic response in skeletal myotubes, see Additional file 1, Figure S8 for a representative Western blot.

## Discussion

The present study is the first to demonstrate that (1) plasma from patients with septic shock induces loss of myosin and activation of the ubiquitin-proteasome pathway and (2) that the atrophic response initiated by plasma from septic shock patients is most severe immediately upon arrival at the ICU and decreases during subsequent days and (3) IL-6 plays a prominent role in inducing the atrophic response.

### Study limitations

The use of cultured C2C12 skeletal myotubes in the current study is highly appropriate to specifically study the contribution of plasma ligands in muscle wasting, since the use of non-diseased muscle tissue virtually excludes any contribution of intrinsic muscle abnormalities. Other *in vitro *models have been used previously by others, including dissected muscle bundles [8,18]. A disadvantage of this latter model is the absence of muscular microcirculation. This induces oxidative stress and limits the supply of ligands to the muscle bundle [19,20]. This may explain why incubation with non-septic human plasma also increased protein degradation in these models [8]. Therefore, we considered that model not suitable for the current research questions.

C2C12 skeletal myotubes reach a considerable degree of differentiation, as indicated by expression of fast twitch skeletal muscle troponin T, alpha-actin and tropomyosin [21]. Permeabilized myotubes generate force when perfused with calcium solutions, with a similar calcium sensitivity of force generation compared to mature skeletal muscle fibers [21]. Nevertheless, data from our own lab [11] and others [22,23] demonstrated differences in some physiological processes compared to mature skeletal muscle, such as intracellular calcium handling. Accordingly, C2C12 cultured myotubes are suitable for addressing specific research questions, but limitations should be recognized.

### Induction of muscle atrophy in septic shock

Sepsis affects skeletal muscle physiology at different stages of excitation-contraction coupling, including contractile protein function, muscle protein content (atrophy), membrane excitability and mitochondrial function [24]. We found that myosin content is reduced by approximately 25% after exposure of muscle to plasma from patients with septic shock. These data are in line with previous studies on rodents, showing a rapid loss of muscle mass upon induction of sepsis [6,25,26]. The triggers that activate muscle proteolysis in early septic shock are largely unknown. Observational studies have proposed several risk factors, including cytokines, corticosteroids, hyperglycemia and immobilization [1]. Yet, as their effects intermingle *in vivo*, it is very difficult to establish which of these risk factors do play a role in skeletal muscle wasting in critically ill patients. By specifically studying the potency of plasma to induce muscle wasting, the current study demonstrates that plasma ligands play a prominent role in inducing muscle proteolysis in patients with septic shock. A previous study found that serum of critically ill patients affect membrane excitability and the excitation-contraction coupling process of isolated muscle fibers [27]. Altogether these findings indicate that circulating factors contribute to the development of muscle weakness in critically ill patients.

We demonstrated that the atrophic response to plasma obtained beyond the day of ICU admission was less prominent, but still present. These findings underscore the necessity of early interventions in the prevention of muscle atrophy in septic shock patients. Since the set-up in the current study specifically addresses the effect of plasma ligands, we do not exclude that other factors, such as immobilization [28] and production of inflammatory mediators by the muscle itself [29,30] also contribute to muscle wasting in septic patients, in particular during prolonged ICU stay.

### Circulating ligands

An important question is which plasma factors in septic shock patients initiate muscle atrophy. First, inflammatory cytokines such as IL-6, TNF-alpha, IL-1-beta and IFN-γ are often implicated in muscle wasting diseases. In our study, the latter three cytokines were below the detection limit in all healthy subjects and the majority of the septic shock patients, suggesting that these were not a major factor in the development of atrophy in our model. Plasma IL-6 levels were elevated in patients with septic shock and plasma levels significantly correlated with the severity of myosin loss. Moreover, blocking IL-6 in plasma from septic shock patients diminished the atrophic response in skeletal myotubes. These data indicate a prominent role of IL-6 in inducing muscle atrophy during septic shock. Noteworthy, the addition of IL-6 to plasma from controls did not induce atrophy of skeletal myotubes, even when a supra-physiological IL-6 concentration of 50 ng/ml was applied. Thus, while IL-6 in plasma from septic shock patients is important to induce severe muscle atrophy, other plasma factors seem to be needed as well. Second, hyperglycemia has been associated with muscle wasting in critically ill patients [31] and hyperglycemia induces protein degradation in cultured muscle [32]. Yet, patients in the current study were normoglycemic (average glucose level 6.8 ± 0.9 mM), as strict glucose control is part of our routine clinical care. Accordingly, it is unlikely that hyperglycemia did directly contribute to muscle wasting in our model. Third, neuromuscular blocking agents have been associated with the development of muscle atrophy [33], although this has been challenged in recent clinical studies [34]. Nevertheless, the last bolus of rocuronium was administered more than four hours before blood withdrawal, ruling out an effect of rocuronium in muscle wasting in our study. Fourth, high doses of corticosteroids have been associated with skeletal muscle wasting [35]. In the current study, 13 out of 21 patients received low dose (maximal one bolus of 100 mg i.v.) hydrocortisone prior to blood withdrawal. No significant difference in atrophy response at Day 1 was observed between patients that had received hydrocortisone and steroid naive patients (Figure 3C). Moreover, at the time more patients had received hydrocortisone (Day 2 to Day 7) the atrophic response was lower than on Day 1. Plasma cortisol concentrations in five of the studied septic shock patients were above normal levels (150 to 700 nM). Yet, these plasma samples provoked similar reductions of myosin in myotubes as plasma samples with normal cortisol levels (data not shown). Finally, metabolic acidosis is known to induce muscle proteolysis by a glucocorticoid-dependent mechanism [36]. Although plasma pH in most patients was acidic, there was no significant relation between plasma pH and myosin concentration (data not shown).

### Intracellular mechanisms

The main focus of this study was to investigate whether myosin loss is triggered by plasma from patients with septic shock, but we also studied activation of proteolysis in these skeletal myotubes. The ubiquitin-proteasome pathway is the main proteolytic system in eukaryotic cells and controls both protein quality and quantity [37]. During the course of this pathway proteins are linked to a chain of ubiquitin molecules under regulation of E3-ligases such as MuRF-1 and MAFBx and, subsequently, recognized and degraded by the proteasome. Recent evidence from our lab indicates that myosin degradation follows this pathway, as proteasome inhibition restores myosin content and muscle function in animal models for respiratory muscle weakness [15]. Moreover, components of the ubiquitin-proteasome pathway are up-regulated in skeletal muscle of septic shock patients [38,39]. The current study adds to these earlier observations as we show that plasma from septic shock patients increases ubiquitinated myosin levels and activates MuRF-1 and MAFBx in skeletal myotubes. MuRF-1 has been shown to specifically ubiquitinate myosin, thereby promoting myosin degradation [40,41]. MuRF-1 expression is under control of the transcription factor NFκB [42]. Indeed, exposure to patient plasma increases the activity of NFκB in myotubes within one hour. MAFbx expression occurs independent from NFκB activity, but is also associated with loss of muscle proteins [43]. Noticeably, in mice overexpression of circulating IL-6 enhances MAFbx mRNA and induces loss of muscle mass [44]. In line with that study, we found that MAFbx expression diminished after Day 2 on the ICU and followed a similar trend as IL-6, which in turn is inversely related to myosin content. Therefore, these data further support the notion that IL-6 is involved in the initiation of skeletal muscle atrophy in septic shock patients.

## Conclusions

The present study demonstrates that plasma from patients with septic shock induces loss of the contractile protein myosin in skeletal myotubes. This atrophic response is most severe to plasma from the early phase of sepsis and is associated with activation of key regulators of proteolysis. IL-6 may play a role in the early development of muscle atrophy in septic shock patients.

## Key messages

• Plasma from patients with septic shock induces loss of the contractile protein myosin in non-diseased muscle

• Plasma-induced myosin loss is most severe in the early phase of sepsis and is associated with activation of key regulators of proteolysis

• IL-6 may play a role in the early development of muscle atrophy in septic shock patients

## Abbreviations

DMEM: Dulbecco's Modified Eagle's Medium; EMSA: electrophoretic mobility shift assay; ICU: intensive care unit; IFN-γ: interferon gamma; IL-1β: interleukin 1 beta; IL-6: interleukin 6; MAFbx: muscle atrophy F-box protein; MuRF-1: muscle RING finger protein-1; NFκB: nuclear factor Kappa B; TNF-α: tumor necrosis factor alpha.

## Competing interests

The authors declare that they have no competing interests.

## Authors' contributions

HvH contributed to designing the study, acquiring, analyzing and interpreting the data, and writing the manuscript. WS participated in acquisition of patient characteristics and contributed to drafting the manuscript. ML and FL carried out the biochemical analyses and contributed to revising the manuscript. JZ and PNRD helped to design the study and contributed to revising the manuscript. RD performed the statistical analyses and contributed to revising the manuscript. JvdH participated in the design of the study, the analysis of the data and contributed to revising the manuscript. LH conceived of the study and participated in its design and coordination, the interpretation of the data and helped to draft the manuscript. All authors read and approved the final manuscript for publication.

## Supplementary Material

Additional file 1 **Online supplement**. The online supplement to this manuscript contains detailed methods descriptions, additional data and pictures of representative Western blots. Click here for file
