# Supplementary material for: Plasma from septic shock patients induces loss of muscle protein
Source: Crit Care. 2011 Sep 29;15(5):R233. doi: 10.1186/cc10475 (PMC3334781; doi:10.1186/cc10475)

Plasma from septic shock patients induces loss of muscle protein

*Online supplement*

H.W.H. van Hees, W.M. Schellekens, M. Linkels, F. Leenders, J. Zoll,

R. Donders, P.N.R. Dekhuijzen, J. G. van der Hoeven and L.M.A. Heunks

*Muscle culture*

Muscle cells, C2C12 myoblasts, were allowed to proliferate in Dulbecco’s modified Eagle’s medium (DMEM) supplemented with 10% fetal calf serum at 37°C in a 5% atmosphere of CO_2_. When cells reached 100% confluence proliferation medium was replaced by DMEM containing 2% Horse Serum, which resulted in differentiation of myoblasts into myotubes. Culture media were refreshed every day. Since the current experimental design requires skeletal myotubes with a stable myosin content, pilot studies were performed to assess the number of days of differentiation needed to obtain myotubes with a stable myosin content. As shown in figure 1, after 7 days of differentiation myosin content was stable. Accordingly, in all subsequent experiments myotubes were used after 7 days of differentiation. In subsequent pilot experiments it was investigated if plasma from controls affects myosin content in skeletal myotubes. To this end, differentiated myotubes were exposed to either plasma from control subjects or differentiation medium for 24 hours. As shown in figure 2, plasma from human control subjects does not affect myosin content.

*Analysis of myosin content*

Myotubes were first washed with PBS and then harvested in 400 μl SDS loading buffer (125 mM Tris pH 8.8, 4% SDS, 40% glycerol, brome phenol blue, 0.1 mM phenyl-methylsulphonylfluoride and 50 mM dithiothreitol). To reduce viscosity, each cell lysate was passed through a needle attached to a plastic syringe. Subsequently, the samples were boiled for 3 minutes. Proteins in the cell lysates were separated by performing electrophoresis on 8% SDS-PAGE gels. After separation, the proteins were transferred by Western blotting to a nitrocellulose membrane. The membrane was stained with antibodies against myosin and tubulin, respectively anti-Myosin Heavy Chain (Bio-connect) and Anti-Bovine α-tubulin (Molecular Probes) in a 1:2000 dilution. Thereafter, the membrane was incubated with a secondary IRDye 800CW Conjugated Goat (polyclonal) Anti-Mouse IgG (GAM-800) antibody (LI-COR). This second antibody was labeled with a fluorescent dye and its intensity was scanned and quantified with the Odyssey infrared imaging system. To correct for different sample loading myosin content was corrected for tubulin content for each sample. Myosin analysis was repeated in duplo to confirm the results.

*Analysis of NFkB activity*

NFkB DNA binding activity was determined by electrophoretic mobility shift assay (EMSA). Nuclear proteins for EMSA were isolated from myotubes as follows. Myotubes were washed in ice-cold PBS, harvested in 2 ml homogenization buffer (10 mM HEPES (pH 7.9), 1.5 mM MgCl2, 10 mM KCl, 0.5 mM DTT and 0.5 mM PMSF) and incubated on ice for 10 minutes. Cell membranes were disrupted using a Dounce homogenizer. The myotubes were centrifuged for 10 minutes at 1000 g at 4˚C. Subsequently the pellet with nuclear extracts was lysed in 80 μl lysis buffer (50 mM HEPES (pH 7.0), 250 mM NaCl, 0.1% NP-40, 5 mM EDTA, 0.5 mM DTT, 1 mM PMSF and 1:1000 μl protease inhibitor cocktail) and disrupted using sonification. To discard the debris the samples were centrifuged for 10 minutes at 17000 g at 4˚C. Nuclear protein content in the supernatant was determined with a Bradford assay kit (Bio-Rad, Veenendaal, the Netherlands). Nuclear extracts (10 µg) were mixed with double stranded oligonucleotides containing an NF-κB consensus binding site (5’-AGTTGAGGGGACTTTCCCAGGC-3’) were radiolabeled with ^32^[P]-ATP using T4 polynucleotide kinase (Promega, Madison, Wisconsin, USA) and incubated at room temperature for 20 min. Then, these samples were loaded on a 4% polyacrylamide gel. After electrophoresis for 45 min., the gel was dried and exposed for ~24h to an X-ray film. The bands on the film quantified by using optical densitometry software (GeneTools, Syngene, Cambridge, UK). Analysis of NFkB activity was repeated in duplo to confirm the results.

*Analysis of E3-ligase mRNA and ubiquitinated myosin levels*

For mRNA analysis skeletal myotubes were washed in PBS and harvested in 500 μl Trizol. After addition of chloroform and centrifugation of the cell lysates at 12000g (4°C) RNA was isolated from the upper aqueous phase by using the RNeasy Mini Kit (Qiagen). The RNA concentration was determined with the NanoDrop 2000c spectrophotometer (Thermo scientific). The purified RNA was transcribed into cDNA with the iScript cDNA Synthesis Kit (Bio-Rad). To determine the expression levels of MAFbx and MuRF-1 a quantitative polymerase chain reaction (Q-PCR) was performed. These expression levels were corrected for expression levels of the housekeeping gene 40S ribosomal protein S9 (RPS9). The following primer sequences were used: MAFbx reverse: 5’-TCA GCC TCT GCA TGA TGT TC-3’, MAFbx forward: 5’-GAC TGG ACT TCT CGA CTG CC-3’, MuRF-1 reverse: 5’-CAA CCT CGT GCC TAC AAG ATG-3’,MuRF-1 forward: 5’-CAA CCT GTG CCG CAA GTG-3’, RPS9 reverse: 5’-CTG CTT GCG GAC CCT AAT GT-3’, RPS9 forward: 5’-CGG CCC GGG AGC TGT TGA CG-3’. For determining the amount of ubiquitinated myosin, diaphragm samples were homogenized in 100 volumes ice-cold buffer pH 7.4 (20mM Tris, 20 mM EGTA, 1mM DTT, protease inhibitor cocktail, 0,5% SDS), boiled for 1 min. and centrifuged at 13,000 x g, 20 °C for 5 min. After protein concentration determination of the resulting supernatants soluble proteins were subjected to routine Western blotting using polyacrylamide SDS-gels and specific antibodies (anti-poly-ubiquitin, Biomol, Exeter, UK; anti-myosin heavy chain; Bio-connect). Thereafter, the membrane was incubated with a horseradish peroxidase conjugated goat anti-mouse antibody (Pierce, Ettenleur, the Netherlands) for subsequent chemiluminiscent detection. Protein bands were quantified using optical densitometry software (GeneTools, Syngene, UK). For each lane, the intensity of the poly-ubiquitin-band was divided by the intensity of the myosin band providing a measure of ubiquitinated myosin per total myosin.

**Tables**

Table s1

Ratios of patients that received specific clinical care during follow-up.

|  | Day 2 | Day 5 | Day 7 |
| --- | --- | --- | --- |
| Ventilator dependent | 8/9 | 6/9 | 5/9 |
| Inotrope/vasopressor use | 7/9 | 1/9 | 1/9 |
| Corticosteroids use | 6/9 | 4/9 | 3/9 |

Legends to the figures

Figure s1

Myosin heavy chain content was measured in myoblastst / myotubes at different levels of differentiation. Myosin content increases with myotube differentiation up to day 7.

Figure s2

Myosin content in skeletal myotubes that were incubated for 24 hours with either normal culture medium (n=9) or plasma from healthy subjects (controls; n=12).

Figure s3

Representative Western blots of myotubes that were either exposed to plasma from controls (n=6) or plasma from patients with a septic shock (n=6). Blots were stained with antibodies directed against myosin (MyHC) and tubulin.

Figure s4

Western blot stained with antibodies directed against myosin (MyHC) and tubulin of plasma samples after incubation on myotubes. The left lane served as a positive control and was loaded with a homogenate of myotubes.

Figure s5

Myosin content in myotubes that were either exposed to plasma from controls (n=12) or plasma from patients with a septic shock that received steroids prior to blood withdrawal (n=13) or steroid naive septic shock patients (n=8). *p < 0.05 vs. controls

Figure s6

Representative electrophoretic mobility shift assay (EMSA) of NFkB activity. The two black bands in each lane reflect two subunits of activated NFkB.

Figure s7

Representative Western blots of myotubes that were either exposed to plasma from patients with a septic shock (n=5), with and without addition of anti-IL-6 (100ng/ml). Blots were stained with antibodies directed against myosin (MyHC) and tubulin.

Figure s8

Representative Western blots of myotubes that were either exposed to plasma from controls (n=2), with addition of IL-6 in different concentrations (0, 200, 600 and 50000 pg/ml). Blots were stained with antibodies directed against myosin (MyHC) and tubulin.

**Figures**

Figure s1


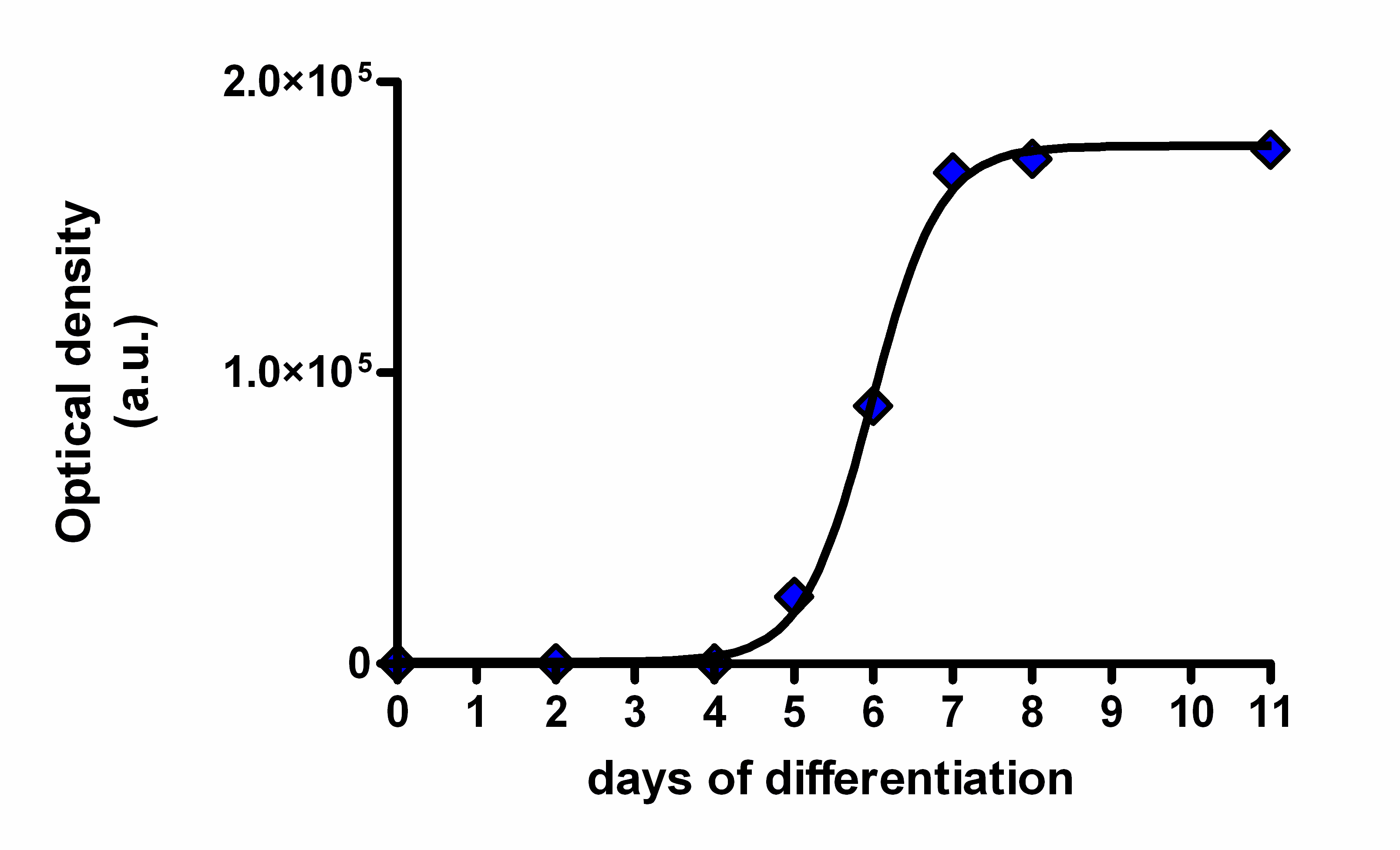


Figure s2


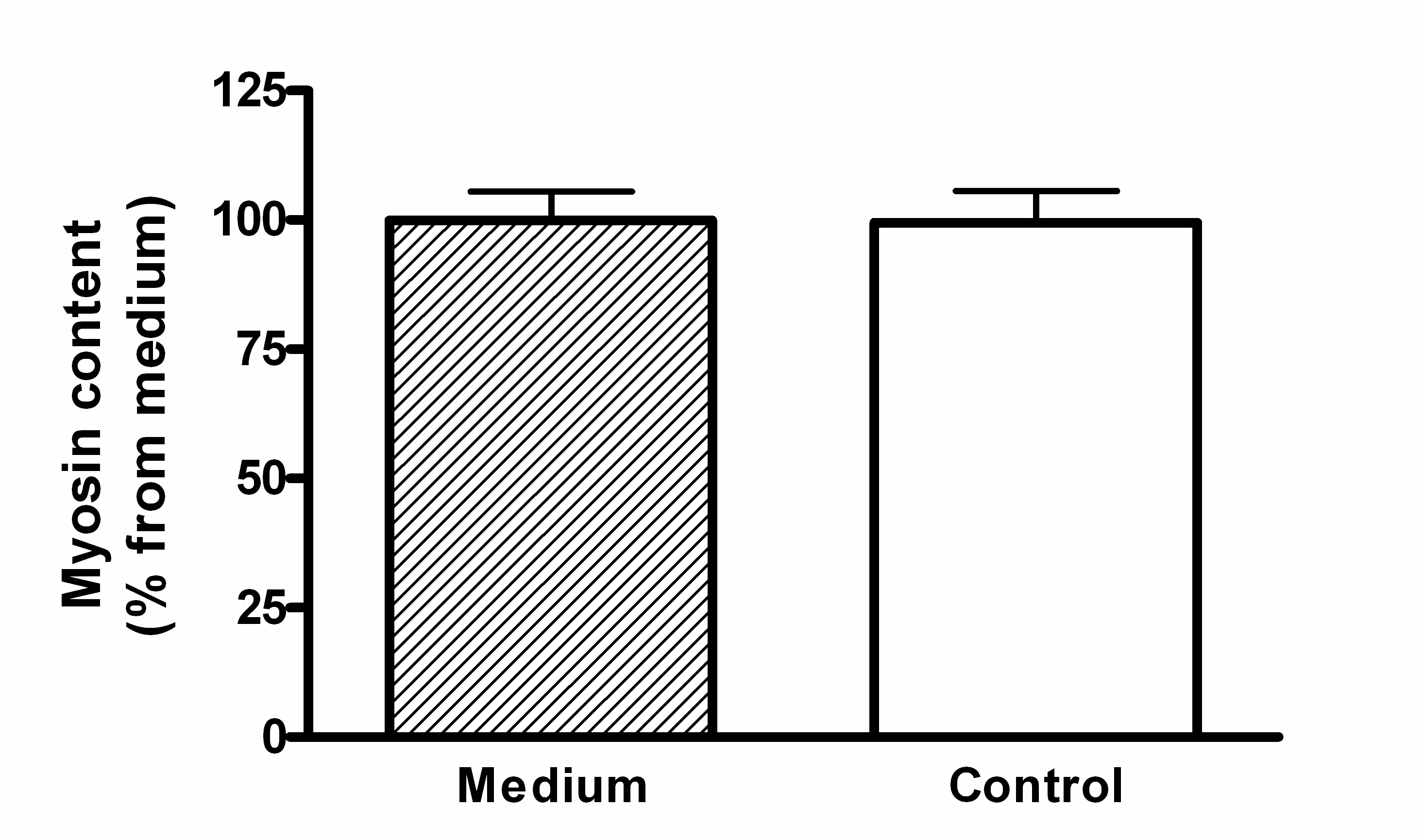


Figure s3


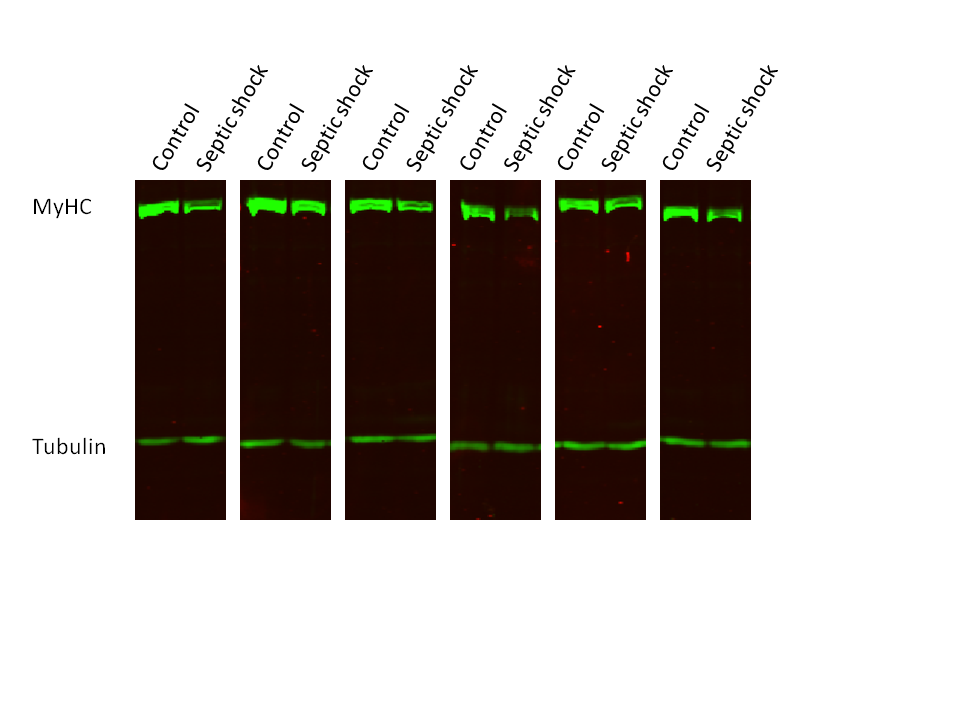


Figure s4


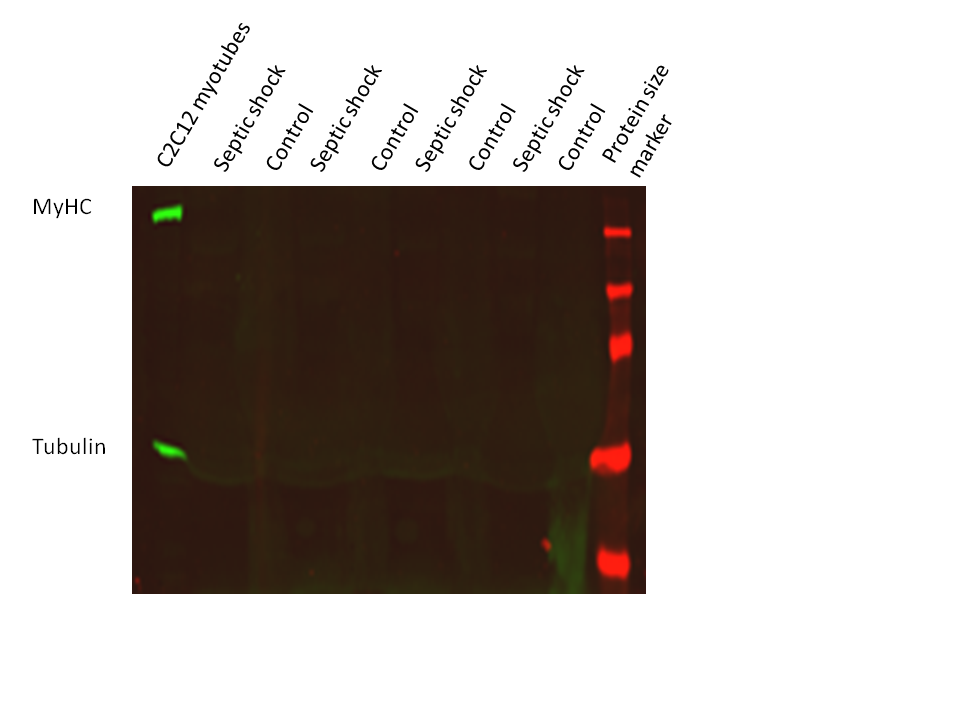


Figure s5


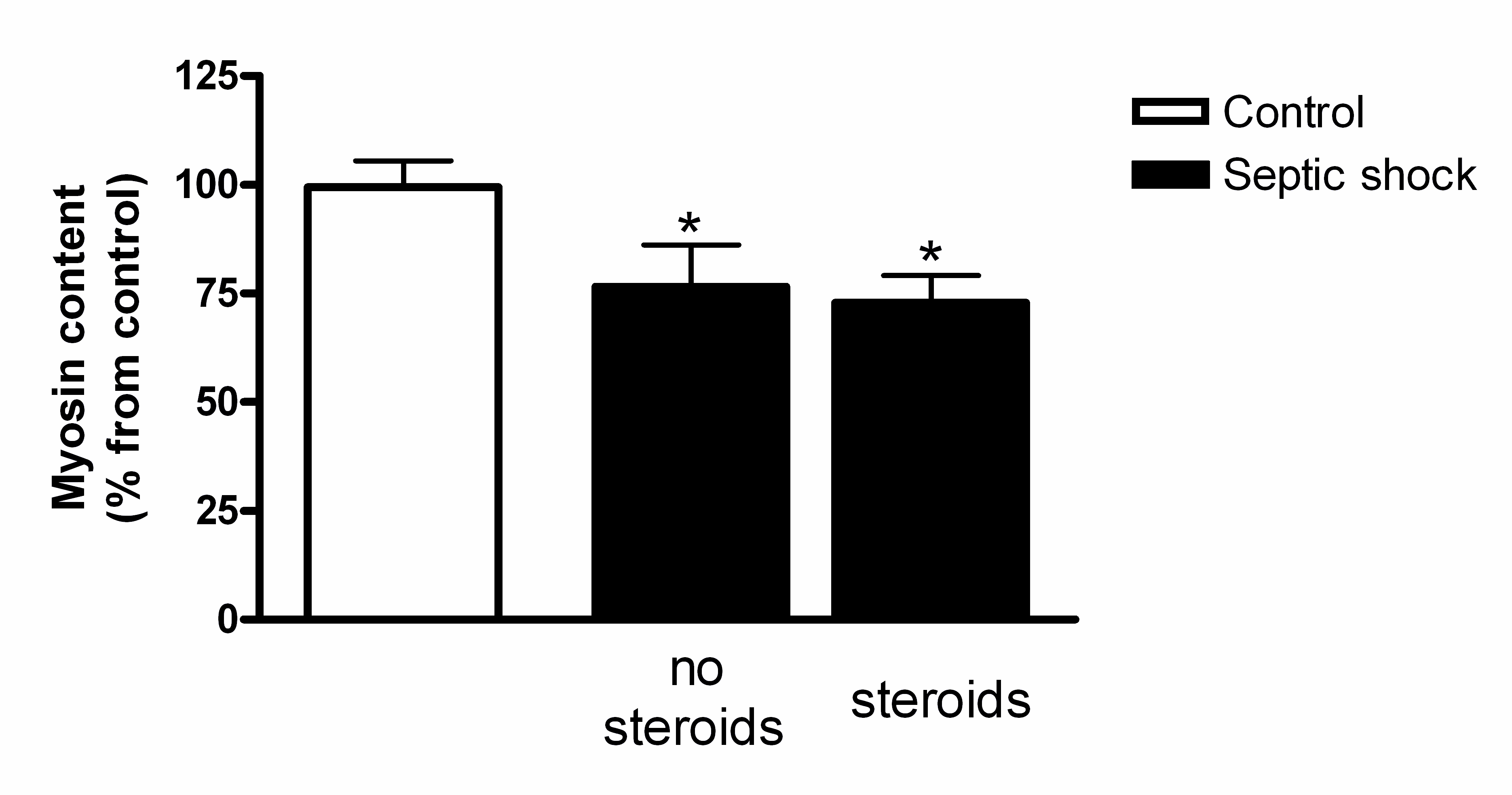


Figure s6


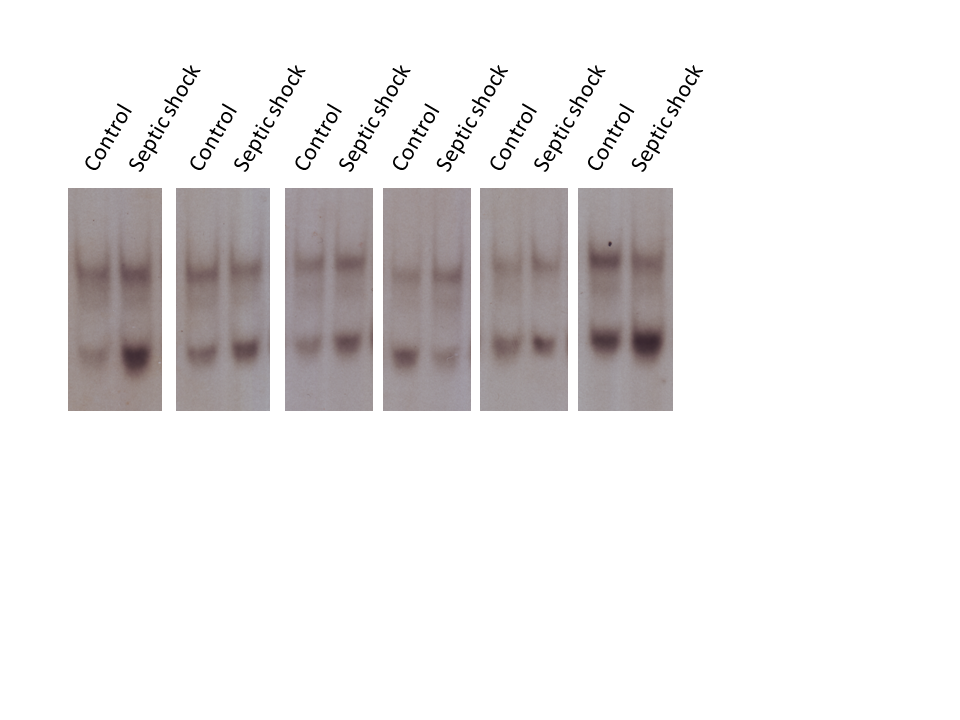


Figure s7


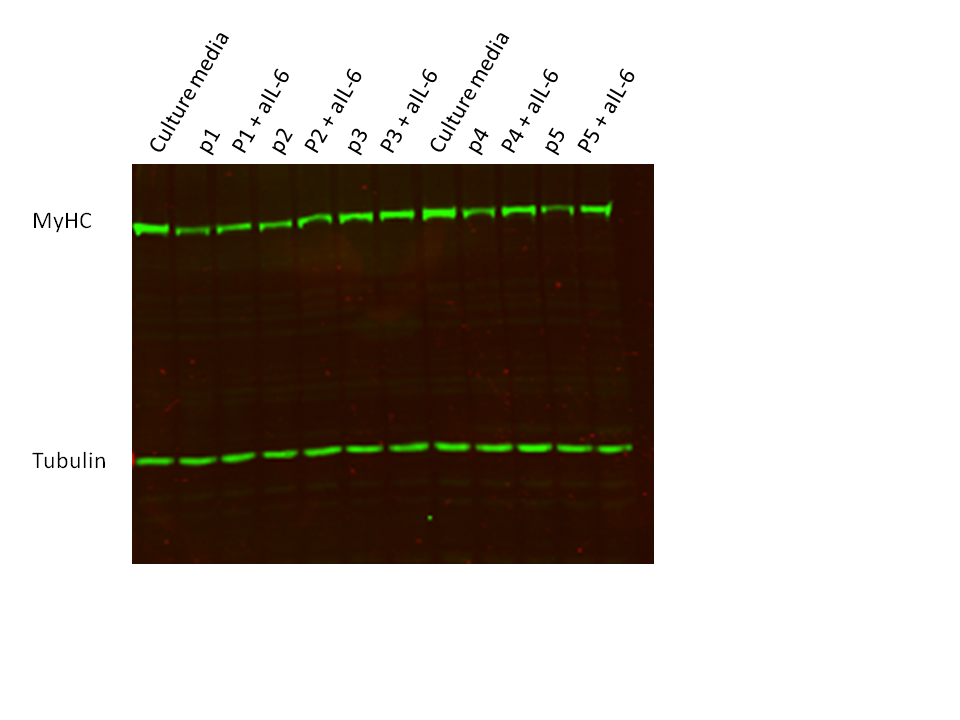


Figure s8


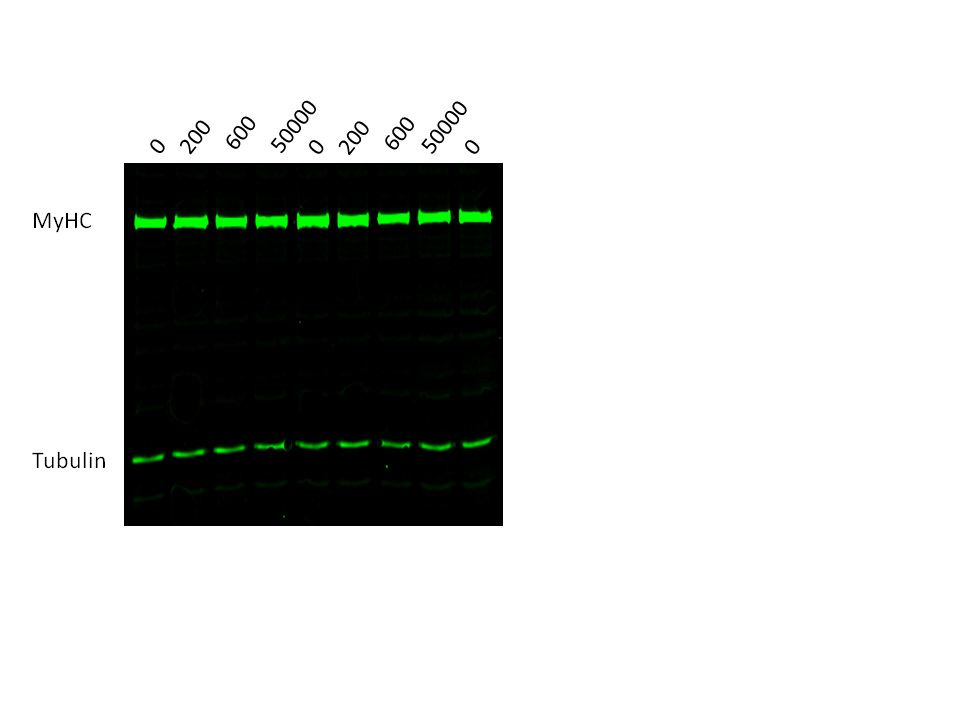

Supplement: Additional file 1 — Online supplement. The online supplement to this manuscript contains detailed methods descriptions, additional data and pictures of representative Western blots. [file cc10475-S1.DOC]
